# Root Canal Transportation and Centering Ability of Nickel-Titanium Rotary Instruments in Mandibular Premolars Assessed Using Cone-Beam Computed Tomography

**DOI:** 10.2174/1874210601711010071

**Published:** 2017-02-14

**Authors:** Iussif Mamede-Neto, Alvaro Henrique Borges, Orlando Aguirre Guedes, Durvalino de Oliveira, Fábio Luis Miranda Pedro, Carlos Estrela

**Affiliations:** 1 Federal University of Goiás Praça Universitária s/n, Setor Universitário, 74605-220 Goiânia, GO, Brazil; 2 University of Cuiaba, Avenida Manoel José de Arruda 3.100, Jardim Europa, 78065-900 Cuiabá, MT, Brazil

**Keywords:** Canal Transportation, CBCT, Centralization, Endodontics, Nickel-titanium Instruments

## Abstract

**Introduction:**

The aim of this study was to evaluate, using cone-beam computed tomography (CBCT), transportation and centralization of different nickel-titanium (NiTi) rotary instruments.

**Methods:**

One hundred and twenty eight mandibular premolars were selected and instrumented using the following brands of NiTi files: WaveOne, WaveOne Gold, Reciproc, ProTaper Next, ProTaper Gold, Mtwo, BioRaCe and RaCe. CBCT imaging was performed before and after root canal preparation to obtain measurements of mesial and distal dentin walls and calculations of root canal transportation and centralization. A normal distribution of data was confirmed by the Kolmogorov-Smirnov and Levene tests, and results were assessed using the Kruskal-Wallis test. Statistical significance was set at 5%.

**Results:**

ProTaper Gold produced the lowest canal transportation values, and RaCe, the highest. ProTaper Gold files also showed the highest values for centering ability, whereas BioRaCe showed the lowest. No significant differences were found across the different instruments in terms of canal transportation and centering ability (P > 0.05).

**Conclusion:**

Based on the methodology employed, all instruments used for root canal preparation of mandibular premolars performed similarly with regard to canal transportation and centering ability.

## INTRODUCTION

Endodontic treatment success depends on proper cleaning, widening, and shaping of the root canal system [1]. Mechanical instrumentation includes both enlargement and shaping, and it is important to enhance the effectiveness of irrigants and antibacterial medicaments in eradicating bacteria and eliminating bacterial by-products [2], thus creating adequate space for three-dimensional obturation [2, 3]. Nickel-titanium (NiTi) rotary files were developed in the 1980s and have been associated with shorter instrumentation time and better cutting efficiency when compared with hand instruments [2]. Conversely, NiTi rotary files have larger tapers and can therefore generate increased friction and stress when compared with hand files [4-6]. Moreover, more severely curved canals may cause increased stress on rotary files and consequently lead to perforations, canal transportation, ledge and zip formation [7], and/or instrument fracture [8, 9].

Instruments and instrumentation techniques should be chosen and combined based on their shaping ability, particularly in curved canals [8], and on the possibility to achieve faster preparations, without deviations [4]. The various NiTi file systems commercially available have different characteristics in terms of their cross-sectional shape, rake angle, taper, depth of flutes, and number of spirals or flutes per unit length – all these conditions may affect file behavior [8,10,11]. For instance, Mtwo instruments have an S-shaped, cross-sectional design and a positive rake angle with 2 cutting edges and low radial contact to increase their flexibility and improve performance inside the root canal [12, 13]. RaCe instruments have a triangular cross-sectional design with sharp, alternating cutting edges that enhance cutting efficiency while producing a more centered canal shape [14-17]. The ProTaper family of instruments comprises NiTi files with a progressively tapered design. ProTaper Next has an off-centered rectangular cross section that gives the file a snake-like swaggering movement as it advances into the root canal. ProTaper Gold has a triangular cross section manufactured by proprietary metallurgy that delivers increased flexibility and resistance to cyclic fatigue [9, 14, 18-21]. Finally, Reciproc and WaveOne, two single-use reciprocating systems commercially available, are fabricated from M-wire alloy, which increases flexibility and improves cyclic fatigue [9, 22-24].

Root canals show variations in cross-sectional anatomy; the apical limit of instrumentation should be determined based on both anatomical aspects and endodontic instrument characteristics [25]. Apical enlargement is beneficial to reduce the extrusion of debris and the presence of remaining bacteria [2, 22]. Conversely, a smaller canal size can reduce susceptibility to tooth fracture [26, 27]. In fact, this aspect remains a very controversial topic in the literature: while Wu *et al.* [28] suggested terminating instrumentation 2-3 mm and 0-2 mm short of the apex, depending on biological conditions, Souza [29] recommended extending instrumentation to 1-2 mm beyond the foramen.

Intraoral periapical radiographs are widely used both in research and in clinical endodontics to aid in diagnosis and management. However, they offer limited, two-dimensional images of an extremely complex anatomical structure [30]. Three-dimensional cone-beam computed tomography (CBCT), in turn, can yield sequential axial images of root canals from the coronal to the apical region, or *vice versa*, and is extremely useful in determining the exact position of anatomic structures, revealing details of the internal root canal anatomy, and helping identify points of communication between root canals and the periodontal space [30, 31]. Because of both its accuracy and the possibility to preserve the tooth structure, CBCT has been increasingly used to evaluate apical transportation and centralization [30-33]. More recently, the use of CBCT images has also been validated in anatomical studies, with the use of sequential 0.110-mm/0.110-mm slices [34].

Considering the importance of correlating instrument characteristics and root canal anatomical aspects to ensure endodontic treatment success [35, 36], the aim of this study was to evaluate, using CBCT, transportation and centralization within the root canal of different NiTi rotary instruments, namely, WaveOne Large (Dentsply Maillefer, Ballaigues, Switzerland), WaveOne Gold Large (Dentsply Maillefer), Reciproc (VDW Dental, Munich, Germany), ProTaper Next (Dentsply Maillefer), ProTaper Gold (Dentsply Maillefer), Mtwo (VDW Dental), BioRaCe (FKG Dentaire, La Chaux de Fonds, Switzerland), and RaCe (FKG Dentaire). The null hypothesis was that large tapered instruments of different brands would present similar results in terms of root canal transportation and centering ability in mandibular premolars.

## MATERIALS AND METHODS

The protocol for this study was approved by the Research Ethics Committee at the Federal University of Goiás, Goiânia, Brazil (CAAE: 53712816.1.0000.5083).

### Tooth Selection and Working Length Determination

A total of 128 mandibular premolars (r > 8 mm) [37] with fully formed apices and root canals were selected for this study. The teeth exhibited no defects, their root canals were not calcified, showed no internal or external root resorption, no prosthetic crowns or dental posts, no signs of prior endodontic treatment, and no aberrant canal morphology; each tooth had a single canal and a single apical foramen, based on buccal and proximal radiographic examinations. To increase standardization, crowns were removed and only teeth measuring 16 mm, confirmed with a digital pachymeter (Starrett Série 799, São Paulo, Brazil), were included in the study.

To detect differences among the groups, sample size was calculated considering α = 0.05, using a statistical software [38]. Teeth were numbered from 1 to 128 and randomly assigned to one of eight groups (n = 16 each) using the same software [38]. The program was set according to the number of specimens in each group (n = 16), the number of groups (n = 8), and the name of each group according to the instruments tested, as follows: WaveOne Large (Dentsply Maillefer), WaveOne Gold Large (Dentsply Maillefer), Reciproc R50 (VDW Dental), ProTaper Next X1 to X5 (Dentsply Maillefer), ProTaper Gold S1 to F5 (Dentsply Maillefer), Mtwo 15/0.05 to 50/0.04 (VDW Dental), BioRaCe BR0 25/0.08 to BR6 50/0.04 (FKG Dentaire), and RaCe 15/0.04 to 50/0.02 (FKG Dentaire).

Standard access cavities were made using round diamond burs #1011 and #1012 (KG Sorensen, Barueri, Brazil) coupled to a high-speed handpiece with air and water spray cooling. The apical patency of all root canals was confirmed using a #10 K-file (Dentsply Maillefer), and canals patent to a size greater than ISO 15 were discarded. Working length was determined using a #15 K-file (Dentsply Maillefer), which was introduced into the root canal until it became visible at the apical foramen, with the aid of an operating microscope (Microscope DM Premium, OPTO, São Paulo, Brazil). Working length was set to 1 mm short of the apex.

### Mechanical Preparation

Root canals were identified under 12x magnification using a DM Premium operating microscope (OPTO) and explored using a #15 K-file (Dentsply Maillefer). During mechanical preparation, roots were involved in gauze and fixed in a vise (Metalsul, Joinville, Brazil). All instruments were driven using the X-Smart Plus (Dentsply Maillefer), in accordance with manufacturers’ instructions – briefly, for ProTaper and Mtwo, speed of 300 rpm and torque of 2 Ncm; for BioRaCe and RaCe, 600 rpm and 1 Ncm, respectively; for WaveOne and Reciproc, parameters are not disclosed by the manufacturer. After each instrument use or after three pecks with the reciprocating files, canals were irrigated with 3 mL of 2.5% sodium hypochlorite (HalexStar, Goiânia, Brazil). Then, the irrigation needle (NaviTip 31ga, Ultradent, South Jordan, UT) was placed 1 mm short of the established working length, and patency was assessed again using a #15 K-file. Each instrument was used to prepare only one root canal. All root canal preparations were completed by a single operator who was a specialist in endodontics with more than 10 years of experience. Final irrigation was performed with 5 mL of 17% EDTA (F&A Laboratório Farmacêutico Ltda., São Paulo, Brazil) for 3 minutes followed by 3 mL of 2.5% sodium hypochlorite.

### Image Capture

Root canal transportation and instrument centralization were measured both before and after mechanical preparation at 3 mm from the radicular vertex. Images of tooth roots were evaluated in three different planes (axial, coronal, and sagittal) in search of synchronization between the first and second sets of images obtained for each specimen, using the synchronization tool of the image processing software (PreXion 3D Viewer, TeraRecon Inc., Foster City, USA). Navigation in the axial plane started at the most extreme point of the root apex and continued for 3 mm. Image visualization was optimized using the software’s magnification, brightness, and contrast adjustment tools.

CBCT images were obtained using a PreXion 3D scanner (PreXion 3D Inc., San Mateo, USA) and the following settings: thickness, 0.100 mm; dimensions, 1.170 mm x 1.570 mm x 1.925 mm; field of view, 56.00 mm; voxel, 0.100 mm, 33.5 seconds (1024 views); tube voltage, 90 kVp; tube current, 4 mA; and exposure time, 33.5 seconds. Images were examined using the scanner’s proprietary software (PreXion 3D Viewer) on an Intel Core 2 Duo-6300 1.86 MHz (Intel Corp, Santa Clara, USA) PC workstation running Windows XP professional SP-2 (Microsoft Corp, Redmond, USA) and equipped with an NVIDIA GeForce 6200 turbo cache video card (NVIDIA Corporation, Santa Clara, USA) and an EIZO-Flexscan S2000 monitor at a resolution of 1600 x 1200 pixels (EIZO NANAO Corp, Hakusan, Japan).

### Evaluation of Canal Transportation and Centering Ability

The technique developed by Gambill *et al.* [30] was used for this purpose. Fig. (**1**) illustrates the measurement of canal areas. Canal transportation corresponds to a deviation of the prepared canal from its natural axis (in millimeters) after instrumentation when compared with pre-treatment measurements. The mean centering ratio indicates the ability of the instrument to stay centered in the canal.

The direction of canal transportation was assessed from the results obtained for each specimen. A negative result indicated transportation toward the distal portion of the root, whereas a positive result indicated transportation toward its mesial portion. A null result indicated the absence of canal transportation. In the assessment of centering ability, a result of 1 indicated perfect centering; the closer the result was to zero, the worse the instrument’s ability to remain centered in the canal.

### Statistical Analysis

The normal distribution of data was confirmed by the Kolmogorov-Smirnov and Levene tests, and the values obtained were assessed using the Kruskal-Wallis test. Statistical significance was set at 5%. Statistical analyses were performed using the Statistical Package for the Social Sciences (SPSS) version 18.0 for Windows (IBM SPSS Inc., Chicago, USA).

## RESULTS

According to Table **1**, ProTaper Gold yielded the lowest root canal transportation values, and RaCe showed the highest values. As regards apical transportation direction, only the ProTaper Next group had a tendency toward transportation to the distal direction. With regard to centering ability, no instrument promoted perfect results (=1.0). ProTaper Gold showed the highest results, whereas the lowest ones were associated with BioRaCe. No statistically significant differences were observed between the instruments assessed (P > 0.05).

Fig. (**2**) illustrates the results found for canal transportation and centering ability for all the instruments assessed.

## DISCUSSION

The present study was based on the hypothesis that large tapered instruments of different brands would not show increased root canal transportation or reduced centering ability in mandibular premolars. Indeed, the results obtained were statistically similar across the instruments assessed, confirming the null hypothesis. The goal of canal preparation is to widen the apical canal, however without weakening the root and thus increasing the risk of tooth fracture [39]. The apical three millimeters of the root canal are considered a critical area, and the reference for apical enlargement to working length continues to be the use of a file three sizes greater than the first file fitting at the apex [40, 41]. Larger apical apertures can contribute to reduce the presence of microorganisms that may lead to and sustain apical periodontitis [39-41]. Complex root canal anatomy is a major challenge to successful endodontic therapy [41]. Successful instrumentation depends on canal morphology, canal wall thickness, and on the size of the instrument used [36, 40]. In oval and flattened canals, such as mandibular premolars, instrumentation is more difficult because of the greater amount of dentin that has to be removed to achieve the desired canal shape [36, 39-41].

The methodology here employed was reproducible, precise and reliable. Moreover, the model used in this study with extracted natural teeth allows real test conditions. Simulated artificial canals are different in terms of microhardness when compared to root dentin, and the effects created by heat generation during instrumentation can affect the instruments’ cutting blades [36]. Ever since CBCT was introduced in dentistry, it has been widely recognized as an accurate, noninvasive tool that allows quantitative and qualitative three-dimensional evaluation of root canals [30, 32, 37]. In the present study, CBCT image technology was used to evaluate canal transportation and centering ability following root canal preparation with different rotary and reciprocating instruments. Scans of 0.110-mm/0.110-mm axial slices were obtained from the coronal to the apical and *vice versa*. This method allowed dynamic visualization and assessment of the specimens before and after instrumentation using pre-established standards, without examiner interference [34, 42].

Considering the direction of canal transportation, except for ProTaper Next, all other systems showed a tendency toward transport to the mesial (outer) direction, with the distal wall acting in antifurcation direction. These results are also in accordance with the literature [33]. It is important to highlight that no canal transportation value was over 0.300 mm, a critical point above which the apical filling ability of the root canal sealer may be compromised [43].

NiTi rotary instruments represent a major evolution in the standardization of canal preparations [5]. Even though all the systems tested in the present study produced some degree of apical transportation, no significant differences were observed across the instruments, which is in line with other studies [9, 17, 22, 26, 40]. Mtwo files are fabricated from conventional NiTi alloy, constant tapered, and usually associated with good maintenance of the original canal curvature [12, 13]. Within the ProTaper family (Universal, Next, and Gold series), ProTaper Gold is enhanced through a proprietary heat treatment technology that results in less canal transportation when compared with ProTaper Universal and ProTaper Next [20]. The good results associated with ProTaper Gold in our study have been reported before [35]. Conversely, Silva *et al.* [36] had different findings, probably as a result of their different methodology (those authors used simulated curved canals). RaCe instruments, in turn, because of their small cross-sectional shape, show improved flexibility; moreover, their alternating cutting edges avoid the screwing effect and allow preparation of curved root canals to larger apical diameters [21, 28]. Also, even though single-file systems (Reciproc and WaveOne) offer different tapers and sizes at the final 3 mm of their tips (D0-D3), and despite their increased flexibility resulting from the presence of M-Wire alloy in their composition [19-23], no differences were observed for these instruments when compared with the others.

With regard to centering ability, none of the instruments tested in the present study remained perfectly centralized within the root canal. No significant differences were observed among the instruments, but the values obtained with ProTaper Gold were closer to 1, suggesting better centering ability. These results can probably be explained by the noncutting tip design of ProTaper Gold, which functions as a guide to allow easy penetration with minimal apical pressure [35].

In summary, this *in vitro* study showed that all the NiTi rotary systems investigated were safe to use, as they produced minimal apical transportation and remained relatively centralized within the root canal. Further studies should be conducted to replicate these findings in real clinical situations.

## CONCLUSION

Based on the results of this *in vitro* study, all the NiTi rotary systems tested performed similarly with regard to canal transportation and centering ability and were able to maintain the original canal curvature in mandibular premolars.

## Figures and Tables

**Fig. (1):**
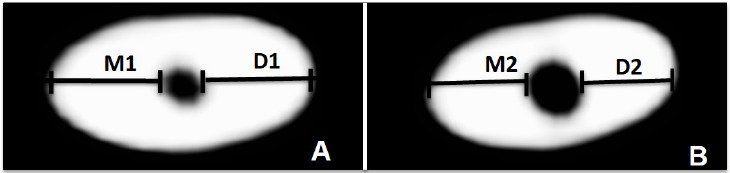
Cross-sectional image illustrating canal area measurements. M1 = shortest distance between mesial aspect of root and mesial portion of non-instrumented canal; M2 = shortest distance between mesial aspect of root and mesial portion of instrumented canal; D1 = shortest distance between distal aspect of root and distal portion of non-instrumented canal; D2 = shortest distance between distal aspect of root and distal portion of instrumented canal.

**Fig. (2):**
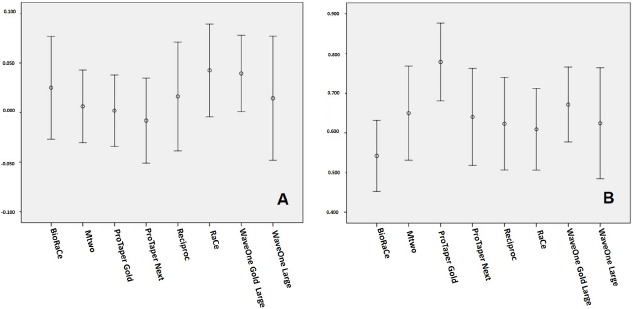
Means and 95% confidence intervals found for **A**) canal transportation and **B**) centering ability with the different instruments assessed.

**Table 1:** Mean ± standard deviation values found for root canal transportation (mm) and centering ability (%) in root canals of mandibular premolars (n=16) following preparation with different instruments.

**Instrument**	**Canal transportation**	**Centering ability**
WaveOne Large	0.014 ± 0.118^a^	0.624 ± 0.263^b^
WaveOne Gold Large	0.039 ± 0.072^a^	0.671 ± 0.177^b^
Reciproc R50	0.016 ± 0.103^a^	0.623 ± 0.219^b^
ProTaper Next	-0.008 ± 0.080^a^	0.640 ± 0.230^b^
ProTaper Gold	0.002 ± 0.068^a^	0.779 ± 0.184^b^
Mtwo	0.006 ± 0.069^a^	0.650 ± 0.223^b^
BioRaCe	0.025 ± 0.097^a^	0.542 ± 0.168^b^
RaCe	0.043 ± 0.088^a^	0.609 ± 0.193^b^

Note: Same superscript letters within each column indicate absence of statistically significant differences between the groups (P > 0.05).
